# Dataset for life cycle assessment of strawberry-package supply chain with considering food loss during transportation

**DOI:** 10.1016/j.dib.2021.107473

**Published:** 2021-10-13

**Authors:** Yuma Sasaki, Takahiro Orikasa, Nobutaka Nakamura, Kiyotada Hayashi, Yoshihito Yasaka, Naoki Makino, Koichi Shobatake, Shoji Koide, Takeo Shiina

**Affiliations:** a Division of Regional Development and Creativity, Graduate School of Arts and Sciences, Iwate University, 3-18-8, Ueda, Morioka, Iwate 020-8550, Japan; b Faculty of Agriculture, Iwate University, 3-18-8, Ueda, Morioka, Iwate 020-8550, Japan; c Food Research Institute, NARO, 2-1-12, Kannondai, Tsukuba, Ibaraki 305-8642, Japan; d Institute for Agro-Environmental Sciences, NARO, 3-1-3, Kannondai, Tsukuba, Ibaraki 305-8604, Japan; e TCO2 Co.,Ltd., 602 Bancho Royal Court, 23-2, Ichiban-cho, Chiyoda, Tokyo 102-0082, Japan; f Graduate School of Horticulture, Chiba University, 648, Matsudo, Chiba, 271-8510, Japan

**Keywords:** Inventory analysis, Life cycle assessment (LCA), Waste and recycle ratio, Packaging, Food loss

## Abstract

This dataset includes two kinds of data (for inventory analysis in Table A1 to A13, and precondition of waste and recycle for plastic and cardboard in Table A14) for conducting life cycle assessment (LCA) of strawberry-package supply chain with considering food loss during transportation Inventory analysis includes input data for LCA analysis. The data in the inventory was referenced from the publication of Plastic Waste Management Institute Plastic Waste Management Institute, (2017) and calculated based on the damage area ratio measured in our co-submitted article (Sasaki et al., 2022). This data helps to reproduce the article (Sasaki et al., 2022) for inventory analysis and re-analyze the environmental impact through the life cycle of strawberry assessed in the co-submitted article. Data of waste (incineration and landfill) and recycle ratios for plastic was collected from the previous reports of the publication Basic Knowledge of Plastic Recycle 2021 (Plastic Waste Management Institute, 2021), and data of the ratios for cardboard was referenced from Transition of Collect Rate on Cardboard (Ministry of the Environment (MOE), 2016). Ratios in this data show Japan-specialized values and is useful for creating the inventory.

## Specifications Table

**Table:** 

Subject	Agriculture engineering
Specific subject area	Life Cycle environmental impact assessment associated with strawberry
Type of data	Table
How data were acquired	Data collection from published reports and calculation of the data in Excel
Data format	Calculated
Parameters for data collection	Our co-submitted article [2] assumed and assessed the domestic supply chain of strawberry in Japan. Thus, all of data in the inventory were collected from literatures focusing on Japanese society, and thus these were Japan-specialized values.
Description of data collection	The input data in the inventory were calculated based on the damage area ratio for each packaging condition at each transportation distance. The ratio was calculated by the vibration test conducted in our co-submitted article [2], and the method of the test was referenced by the article. The input data were the calculated value needed to transport 1 kg of no damaged strawberries to retail. Thus, the input value increases with increase of the damage area ratio because additional strawberries and packaging materials were produced to compensate for the losses caused during transportation by truck.
Data source location	Institution: The National Agriculture and Food Research Organization (NARO) (for vibration test) City/Town/Region: Tsukuba/Ibaraki (which was the region cultivation strawberry in this dataset) Country: Japan Latitude and longitude (and GPS coordinates, if possible) for collected samples/data: E140°05′ and N36°02′
Data accessibility	With the article
Related research article	Sasaki Y., Orikasa T., Nakamura N., Hayashi K., Yasaka Y., Makino N., Shobatake K., Koide S., Shiina T. Optimal packaging for strawberry transportation: evaluation and modeling of the relationship between food loss reduction and environmental impact. Journal of Food Engineering. https://doi.org/10.1016/j.jfoodeng.2021.110767

## Value of the Data

• The life cycle inventory data submitted to the Data in Brief are important to ensure more transparency in the LCA modelling carried out in the co-submitted article [2].
• These data include beneficial information for LCA analysts studying food loss of fruits and vegetable to assess the environmental impact though the life cycle of the products with considering the influence of food loss prevention by packaging on environment.
• These data can be used to discuss a higher-optimized packaging (an ideal packaging), which leads to low food losses and environmental loads, through reproducing the LCA study (co-submitted article [2]) based on parameters in the data.

## 1 Data Description

Tables A1 to A13 show material and energy inputs per mass-based functional unit (kg of undamaged strawberries) for packaging 1, 2, 3, and no-cushioning condition. Input data associated with transportation distance of 0, 50, 100, 500, 1000, and 2000 km and the life cycle stages of strawberry from cultivation to waste are separately shown in each table. Table A14 shows the waste and recycle ratios for package and cardboard. Details of each table were as follows.

**Table A1:** Material and energy inputs per mass-based functional unit (kg of undamaged strawberries) in the three packaging conditions and one no-cushioning condition from (1) cultivation to (3) transportation to the wholesale market (transportation distance: 0 km).

Process	Unit	Packaging 1	Packaging 2	Packaging 3	No-cushioning
(1) Cultivation					

Strawberry	kg	1.00	1.00	1.00	1.00

(2) Package production					

Cardboard	kg	0.40	0.25	0.31	0.25
Package	kg	0.016	0.033	0.065	
OPP film	kg		0.0041	0.0038	
PE bubble wrap	kg	0.013			

(3) Transportation from a fruit sorting facility to the wholesale market					

4 t truck	kg	1.43	1.29	1.38	1.25

Table A1, which includes material and energy inputs per mass-based functional unit (kg of undamaged strawberries) in the three packaging conditions and one no-cushioning condition from cultivation to transportation to the wholesale market (transportation distance: 0 km).

Table A2, which includes material and energy inputs per mass-based functional unit (kg of undamaged strawberries) in the three packaging conditions and one no-cushioning condition from waste transportation (due to food loss) to package waste (due to food loss) (transportation distance: 0 km). Only amounts of package waste associated with incineration and landfilling are shown in Table A2; the residual amounts were recycled.

**Table A2:** Material and energy inputs per mass-based functional unit (kg of undamaged strawberries) in the three packaging conditions and one no-cushioning condition from (5) waste transportation (due to food loss) to (7) package waste (due to food loss) (transportation distance: 0 km).

Process	Unit	Packaging 1	Packaging 2	Packaging 3	No-cushioning
(5) Waste transportation (due to food loss)					

2 t truck	Kg	0.00	0.00	0.00	0.00

(6) Strawberry waste (due to food loss)					

Incineration	Kg	0.00	0.00	0.00	0.00

(7) Package waste (due to food loss) (Incineration and landfill) *					

Cardboard	G	0.00	0.00	0.00	0.00
Package	G	0.00	0.00	0.00	
OPP film	G		0.00	0.00	
PE bubble wrap	G	0.00			

⁎ Only amounts of package waste associated with incineration and landfilling are shown in Table A2; the residual amounts were recycled.

Table A3, which includes material and energy inputs per mass-based functional unit (kg of undamaged strawberries) in the three packaging conditions and one no-cushioning condition from transportation from the wholesale market to package waste (after consumption) (transportation distance: 0 km–2000 km). The inventories in Table A3 were the same for the distance of 0–2000 km because these inventories from waste transportation to package waste (after consumption) were not affected by the transport distance. Only amounts of package waste associated with incineration and landfilling are shown in Table A3; the residual amounts were recycled.

**Table A3:** Material and energy inputs per mass-based functional unit (kg of undamaged strawberries) in the three packaging conditions and one no-cushioning condition from (4) transportation from the wholesale market to (10) package waste (after consumption) (transportation distance: 0 km–2000 km).

Process	Unit	Packaging 1*	Packaging 2*	Packaging 3*	No-cushioning*
(4) Transportation from the wholesale market to retail					

4 t truck	kg	1.43	1.29	1.38	1.25

(8) Waste transportation (after consumption)					

2 t truck	kg	0.45	0.31	0.40	0.27

(9) Strawberry waste (inedible parts)					

Incineration	kg	0.020	0.020	0.020	0.020

(10-1) Package waste (after consumption) (incineration) ⁎⁎					

Cardboard	g	1.3	0.83	1.0	0.83
Package	g	2.3	4.6	9.1	
OPP film	g		0.57	0.53	
PE bubble wrap	g	1.9			

(10-2) Package waste (after consumption) (landfill) ⁎⁎					

Cardboard	g	0.00	0.00	0.00	0.00
Package	g	0.82	1.6	3.3	
OPP film	g		0.21	0.19	
PE bubble wrap	g	0.67			

⁎ The inventories in Table A3 were the same for the distance of 0–2000 km because these inventories from waste transportation to package waste (after consumption) were not affected by the transport distance.

⁎⁎ Only amounts of package waste associated with incineration and landfilling are shown in Table A3; the residual amounts were recycled.

Table A4, which includes material and energy inputs per mass-based functional unit (kg of undamaged strawberries) in the three packaging conditions and one no-cushioning condition from cultivation to transportation to the wholesale market (transportation distance: 50 km).

**Table A4:** Material and energy inputs per mass-based functional unit (kg of undamaged strawberries) in the three packaging conditions and one no-cushioning condition from (1) cultivation to (3) transportation to the wholesale market (transportation distance: 50 km).

Process	Unit	Packaging 1	Packaging 2	Packaging 3	No-cushioning
(1) Cultivation					

Strawberry	kg	1.01	1.01	1.04	1.06

(2) Package production					

Cardboard	kg	0.40	0.25	0.32	0.27
Package	kg	0.017	0.033	0.068	
OPP film	kg		0.0041	0.0040	
PE bubble wrap	kg	0.013			

(3) Transportation from a fruit sorting facility to the wholesale market					

4 t truck	kg	1.44	1.30	1.43	1.33

Table A5, which includes material and energy inputs per mass-based functional unit (kg of undamaged strawberries) in the three packaging conditions and one no-cushioning condition from waste transportation (due to food loss) to package waste (due to food loss) (transportation distance: 50 km). Only amounts of package waste associated with incineration and landfilling are shown in Table A5; the residual amounts were recycled.

**Table A5:** Material and energy inputs per mass-based functional unit (kg of undamaged strawberries) in the three packaging conditions and one no-cushioning condition from (5) waste transportation (due to food loss) to (7) package waste (due to food loss) (transportation distance: 50 km).

Process	Unit	Packaging 1	Packaging 2	Packaging 3	No-cushioning
(5) Waste transportation (due to food loss)					

2 t truck	Kg	0.011	0.011	0.052	0.073

(6) Strawberry waste (due to food loss)					

Incineration	Kg	0.0074	0.0087	0.037	0.058

(7-1) Package waste (due to food loss) (incineration) *					

Cardboard	G	0.010	0.0072	0.039	0.049
Package	g	0.0060	0.014	0.12	
OPP film	g		0.0018	0.0071	
PE bubble wrap	g	0.0049			

(7-2) Package waste (due to food loss) (landfill) *					

Cardboard	g	0.000	0.00	0.00	0.00
Package	g	0.010	0.023	0.20	
OPP film	g		0.0028	0.011	
PE bubble wrap	g	0.0078			

⁎ Only amounts of package waste associated with incineration and landfilling are shown in Table A5; the residual amounts were recycled.

Table A6, which includes material and energy inputs per mass-based functional unit (kg of undamaged strawberries) in the three packaging conditions and one no-cushioning condition from cultivation to transportation to the wholesale market (transportation distance: 100 km).

**Table A6:** Material and energy inputs per mass-based functional unit (kg of undamaged strawberries) in the three packaging conditions and one no-cushioning condition from (1) cultivation to (3) transportation to the wholesale market (transportation distance: 100 km).

Process	Unit	Packaging 1	Packaging 2	Packaging 3	No-cushioning
(1) Cultivation					

Strawberry	kg	1.01	1.02	1.04	1.09

(2) Package production					

Cardboard	kg	0.40	0.26	0.33	0.27
Package	kg	0.017	0.034	0.068	
OPP film	kg		0.0042	0.0040	
PE bubble wrap	kg	0.013			

(3) Transportation from a fruit sorting facility to the wholesale market					

4 t truck	kg	1.44	1.32	1.44	1.36

Table A7, which includes material and energy inputs per mass-based functional unit (kg of undamaged strawberries) in the three packaging conditions and one no-cushioning condition from waste transportation (due to food loss) to package waste (due to food loss) (transportation distance: 100 km). Only amounts of package waste associated with incineration and landfilling are shown in Table A7; the residual amounts were recycled.

**Table A7:** Material and energy inputs per mass-based functional unit (kg of undamaged strawberries) in the three packaging conditions and one no-cushioning condition from (5) waste transportation (due to food loss) to (7) package waste (due to food loss) (transportation distance: 100 km)

Process	Unit	Packaging 1	Packaging 2	Packaging 3	No-cushioning
(5) Waste transportation (due to food loss)					

2 t truck	kg	0.011	0.030	0.060	0.11

(6) Strawberry waste (due to food loss)					

Incineration	kg	0.0075	0.023	0.044	0.085

(7-1) Package waste (due to food loss) (incineration) *					

Cardboard	g	0.0098	0.019	0.045	0.071
Package	g	0.0061	0.038	0.14	
OPP film	g		0.0047	0.0083	
PE bubble wrap	g	0.0050			

(7-2) Package waste (due to food loss) (landfill) *					

Cardboard	g	0.00	0.00	0.00	0.00
Package	g	0.010	0.060	0.23	
OPP film	g		0.0075	0.013	
PE bubble wrap	g	0.0080			

⁎ Only amounts of package waste associated with incineration and landfilling are shown in Table A7; the residual amounts were recycled.

Table A8, which includes material and energy inputs per mass-based functional unit (kg of undamaged strawberries) in the three packaging conditions and one no-cushioning condition from cultivation to transportation to the wholesale market (transportation distance: 500 km).

**Table A8:** Material and energy inputs per mass-based functional unit (kg of undamaged strawberries) in the three packaging conditions and one no-cushioning condition from (1) cultivation to (3) transportation to the wholesale market (transportation distance: 500 km).

Process	Unit	Packaging 1	Packaging 2	Packaging 3	No-cushioning
(1) Cultivation					

Strawberry	kg	1.04	1.05	1.16	1.32

(2) Package production					

Cardboard	kg	0.41	0.27	0.36	0.33
Package	kg	0.017	0.035	0.076	
OPP film	kg		0.0043	0.0044	
PE bubble wrap	kg	0.014			

(3) Transportation from a fruit sorting facility to the wholesale market					

4 t truck	kg	1.48	1.36	1.60	1.65

Table A9, which includes material and energy inputs per mass-based functional unit (kg of undamaged strawberries) in the three packaging conditions and one no-cushioning condition from waste transportation (due to food loss) to package waste (due to food loss) (transportation distance: 500 km). Only amounts of package waste associated with incineration and landfilling are shown in Table A9; the residual amounts were recycled.

**Table A9:** Material and energy inputs per mass-based functional unit (kg of undamaged strawberries) in the three packaging conditions and one no-cushioning condition from (5) waste transportation (due to food loss) to (7) package waste (due to food loss) (transportation distance: 500 km).

Process	Unit	Packaging 1	Packaging 2	Packaging 3	No-cushioning

(5) Waste transportation (due to food loss)					

2 t truck	kg	0.050	0.069	0.22	0.40

(6) Strawberry waste (due to food loss)					

Incineration	kg	0.035	0.054	0.16	0.32

(7-1) Package waste (due to food loss) (incineration) *					

Cardboard	g	0.047	0.045	0.17	0.27
Package	g	0.029	0.088	0.53	
OPP film	g		0.011	0.031	
PE bubble wrap	g	0.024			

(7-2) Package waste (due to food loss) (landfill) *					

Cardboard	g	0.00	0.00	0.00	0.00
Package	g	0.046	0.14	0.84	
OPP film	g		0.018	0.049	
PE bubble wrap	g	0.038			

⁎ Only amounts of package waste associated with incineration and landfilling are shown in Table A9; the residual amounts were recycled.

Table A10, which includes material and energy inputs per mass-based functional unit (kg of undamaged strawberries) in the three packaging conditions and one no-cushioning condition from cultivation to transportation to the wholesale market (transportation distance: 1000 km).

**Table A10:** Material and energy inputs per mass-based functional unit (kg of undamaged strawberries) in the three packaging conditions and one no-cushioning condition from (1) cultivation to (3) transportation to the wholesale market (transportation distance: 1000 km).

Process	Unit	Packaging 1	Packaging 2	Packaging 3	No-cushioning
(1) Cultivation					

Strawberry	kg	1.02	1.07	1.21	1.57

(2) Package production					

Cardboard	kg	0.41	0.27	0.38	0.40
Package	kg	0.017	0.035	0.079	
OPP film	kg		0.0044	0.0046	
PE bubble wrap	kg	0.014			

(3) Transportation from a fruit sorting facility to the wholesale market					

4 t truck	kg	1.46	1.38	1.68	1.97

Table A11, which includes material and energy inputs per mass-based functional unit (kg of undamaged strawberries) in the three packaging conditions and one no-cushioning condition from waste transportation (due to food loss) to package waste (due to food loss) (transportation distance: 1000 km). Only amounts of package waste associated with incineration and landfilling are shown in Table A11; the residual amounts were recycled.

**Table A11:** Material and energy inputs per mass-based functional unit (kg of undamaged strawberries) in the three packaging conditions and one no-cushioning condition from (5) waste transportation (due to food loss) to (7) package waste (due to food loss) (transportation distance: 1000 km).

Process	Unit	Packaging 1	Packaging 2	Packaging 3	No-cushioning
(5) Waste transportation (due to food loss)					

2 t truck	kg	0.026	0.086	0.30	0.72

(6) Strawberry waste (due to food loss)					

Incineration	kg	0.018	0.067	0.21	0.57

(7-1) Package waste (due to food loss) (incineration) *					

Cardboard	G	0.024	0.055	0.22	0.48
Package	G	0.015	0.11	0.70	
OPP film	G		0.014	0.041	
PE bubble wrap	G	0.012			

(7-2) Package waste (due to food loss) (landfill) *					

Cardboard	G	0.00	0.00	0.00	0.00
Package	G	0.024	0.17	1.1	
OPP film	G		0.022	0.065	
PE bubble wrap	G	0.019			

⁎ Only amounts of package waste associated with incineration and landfilling are shown in Table A11; the residual amounts were recycled.

Table A12, which includes material and energy inputs per mass-based functional unit (kg of undamaged strawberries) in the three packaging conditions and one no-cushioning condition from cultivation to transportation to the wholesale market (transportation distance: 2000 km).

**Table A12:** Material and energy inputs per mass-based functional unit (kg of undamaged strawberries) in the three packaging conditions and one no-cushioning condition from (1) cultivation to (3) transportation to the wholesale market (transportation distance: 2000 km).

Process	Unit	Packaging 1	Packaging 2	Packaging 3	No-cushioning
(1) Cultivation					

Strawberry	kg	1.09	1.10	1.38	2.10

(2) Package production					

Cardboard	kg	0.44	0.28	0.43	0.53
Package	kg	0.018	0.036	0.090	
OPP film	kg		0.0045	0.0053	
PE bubble wrap	kg	0.015			

(3) Transportation from a fruit sorting facility to the wholesale market					

4 t truck	kg	1.56	1.42	1.91	2.63

Table A13, which includes material and energy inputs per mass-based functional unit (kg of undamaged strawberries) in the three packaging conditions and one no-cushioning condition from waste transportation (due to food loss) to package waste (due to food loss) (transportation distance: 2000 km). Only amounts of package waste associated with incineration and landfill are shown in Table A13; the residual amounts were recycled.

**Table A13:** Material and energy inputs per mass-based functional unit (kg of undamaged strawberries) in the three packaging conditions and one no-cushioning condition from (5) waste transportation (due to food loss) to (7) package waste (due to food loss) (transportation distance: 2000 km).

Process	Unit	Packaging 1	Packaging 2	Packaging 3	No-cushioning

(5) Waste transportation (due to food loss)					

2 t truck	kg	0.13	0.13	0.53	1.4

(6) Strawberry waste (due to food loss)					

Incineration	kg	0.092	0.10	0.38	1.1

(7-1) Package waste (due to food loss) (incineration) *					

Cardboard	g	0.12	0.082	0.39	0.91
Package	g	0.076	0.16	1.2	
OPP film	g		0.020	0.072	
PE bubble wrap	g	0.061			

(7-2) Package waste (due to food loss) (landfill) *					

Cardboard	g	0.00	0.00	0.00	0.00
Package	g	0.12	0.26	2.0	
OPP film	g		0.032	0.12	
PE bubble wrap	g	0.10			

⁎ Only amounts of package waste associated with incineration and landfill are shown in Table A13; the residual amounts were recycled.

Table A14, which includes the waste (incineration and landfill) and recycle ratios for plastic and cardboard in Japan. These ratios were used to calculate the input data in Tables A1 to A13.

**Table A14:** Waste and recycle ratios for plastic and cardboard.

	Incineration	Landfill	Recycle
Plastic (general waste)	14	5.0	81
Plastic (industrial waste)	5.0	8.0	87
Cardboard	0.33	0.0	96.7
		Unit: (%)	

## 2 Experimental Design, Materials and Methods

All the input data were categorized based on the life cycle stages of strawberry transportation, and the stages were as follows; Cultivation, Package production, Transportation from a fruit sorting facility to the wholesale market, Transportation from the wholesale market to retail, Waste transportation (due to food loss), Strawberry waste (due to food loss), Package waste (due to food loss), Waste transportation (after consumption), Strawberry waste (inedible parts), and Package waste (after consumption) stages. It is assumed that food losses are caused only during the transportation stage from a fruit sorting facility to the wholesale market because the purpose of this study is to assess the influence of food loss prevention by packaging during transportation on environment.

All the data were calculated based on needed amount of strawberries and the materials for transporting 1 kg of undamaged products to retail with considering the food losses (damage area ratio) caused during transportation; i.e., the values in Tables A1 to A13 were varied with the damage area ratio because additional strawberries and materials are required to compensate for the losses. For example, if a food loss ratio was 20%, the compensation to cover the loss (to fulfil the functional unit of 1 kg) was cultivating 1.25 kg of strawberries. After the cultivation, 1 kg of undamaged products was to retail and 0.25 kg of them were wasted. The damage area ratio was calculated by an evaluation method referenced by a previous report [5] after a vibration test. The ratio was a proportion of the damage area (dimension) on the strawberry surface after the test to the area before the test. The calculated ratios were shown in the co-submitted article. The vibration test was conducted to obtain the damage area ratio by referencing a previous report [2,6] with a three-dimensional vibration testing machine (FVH146, Saginomiya seisakusho Inc., Japan) in the National Agriculture and Food Research Organization (NARO) in Japan. A random vibration test was conducted with a referenced vibration data (power spectrum density, PSD). This test was regarded by the Japanese Industrial Standards Committee [7] as the best method to reproduce the actual characteristics of vibration and shock (e.g. acceleration) for the packed products during truck transportation. The PSD was measured by Suda et al. [8]. They measured it in a truck condition at a speed of 80 km/h on an expressway in Japan. Thus, transportation distances (0 km to 2000 km) were determined by multiplying the testing time needed for the distance and 80 km/h, e.g., the distance of 500 km was simulated by testing (vibrating) strawberries for 6.25 h. The temperature and humidity during the test were 20°C and not controlled, respectively. This was determined as one of the assumed atmospheric conditions during strawberry transportation in Japan [6]. A transportation distance from wholesale market to retail and a waste transportation for strawberries and packages were set to 50 km based on the reference [6].

Information of the weight of each package and cardboard (materials) and the damage area ratio were referenced from the previous report published by Plastic Waste Management Institute [1]. The input amount of strawberry waste (inedible parts) was referenced from a previous report [9]. This report showed that the ratio of inedible parts, e.g., strawberry calyx, was 2%, and thus the amount of strawberry waste was 0.02 kg in the tables (1 kg of undamaged products were consumed and 2% of them (1 kg*0.02 = 0.02 kg) were wasted). Input amounts in all waste stage were calculated based on the ratios in Table A14. The ratios in this table were referenced from the previous publication [3] for incineration and landfill, and [4] for recycle.

Some assumptions were needed to complete the inventory and the assumptions were described below. Manufacturing agricultural equipment, some facilities (e.g., fruit sorting machine), truck, and pallets used during strawberry transportation were excluded from the input data because environmental impacts associated with the production process of them were considered negligible when conducing LCA, i.e., these impacts are allocated by the usable years of these products when calculating the needed amounts for transportation of 1 kg of strawberry, and thus these impacts are negligibly decreased. More details of this decrease were shown in a previous report [10]. The process for strawberry consumption was also excluded because consuming strawberries does not require cooking or preparation, and thus this process causes no environmental impact. The amounts of recycled materials were not shown in the tables because the influence of recycling on environment was not assessed in our co-submitted article. Transportation of packed strawberries to the wholesale market or retail was conducted with a 4 t truck and waste transportation of them was with a 2 t truck. These were referenced by the previous study [6].

## Ethics Statement

Not applicable.

## Contents

Table A1 to A13 Inventory analysis.

Table A14 Waste and recycle ratios for plastic and cardboard.

## CRediT authorship contribution statement

**Yuma Sasaki:** Writing – original draft, Software, Data curation, Validation, Formal analysis, Investigation. **Takahiro Orikasa:** Conceptualization, Methodology, Software, Supervision, Project administration, Funding acquisition, Writing – review & editing. **Nobutaka Nakamura:** Investigation, Data curation. **Kiyotada Hayashi:** Investigation, Data curation. **Yoshihito Yasaka:** Investigation, Data curation. **Naoki Makino:** Investigation, Data curation. **Koichi Shobatake:** Investigation, Data curation. **Shoji Koide:** Methodology. **Takeo Shiina:** Conceptualization, Methodology.

## Declaration of Competing Interest

The authors declare that they have no known competing financial interests or personal relationships which have or could be perceived to have influenced the work reported in this article.
